# Characterization and diagnosis spectrum of patients with cerebrospinal fluid pleocytosis

**DOI:** 10.1007/s15010-023-02087-8

**Published:** 2023-09-01

**Authors:** Susanne Dyckhoff-Shen, Jan P. Bewersdorf, Nina C. Teske, Stefanie Völk, Hans-Walter Pfister, Uwe Koedel, Matthias Klein

**Affiliations:** 1 Department of Neurology, LMU University Hospital, LMU Munich (en.), Marchioninistr. 15, 81377 Munich, Germany; 2 Department of Medicine, Leukemia Service, Memorial Sloan Kettering Cancer Center, New York, NY USA; 3 Department of Neurosurgery, LMU University Hospital, LMU Munich (en.), Munich, Germany; 4 Emergency Department, LMU University Hospital, LMU Munich (en.), Munich, Germany

**Keywords:** CSF, Pleocytosis, Bacterial meningitis, Score

## Abstract

**Purpose:**

There is an overlap in the cerebrospinal fluid (CSF) characteristics of patients presenting with different etiologies of CSF pleocytosis. Here, we characterized patients with CSF pleocytosis treated in a large hospital.

**Methods:**

A retrospective cohort study of 1150 patients with an elevated CSF leukocyte count > 5 cells/µl treated at a university hospital in Germany from January 2015 to December 2017 was performed. Information on clinical presentation, laboratory parameters, diagnosis and outcome was collected. Clinical and laboratory features were tested for their potential to differentiate between bacterial meningitis (BM) and other causes of CSF pleocytosis.

**Results:**

The most common etiologies of CSF pleocytosis were CNS infections (34%: 20% with detected pathogen, 14% without), autoimmune (21%) and neoplastic diseases (16%). CSF cell count was higher in CNS infections with detected pathogen (median 82 cells/µl) compared to autoimmune (11 cells/µl, *p* = 0.001), neoplastic diseases (19 cells/µl, *p* = 0.01) and other causes (11 cells/µl, *p* < 0.001). The CHANCE score was developed to differentiate BM from other causes of CSF pleocytosis: Multivariate regression revealed that CSF cell count > 100 cells/µl, CSF protein > 100 mg/dl, CRP > 5 mg/dl, elevated white blood cell count, abnormal mental status and nuchal rigidity are important indicators. The CHANCE score identified patients with BM with high sensitivity (92.1%) and specificity (90.9%) (derivation cohort: AUC: 0.955, validation cohort: AUC: 0.956).

**Conclusion:**

Overall, the most common causes for CSF pleocytosis include infectious, neoplastic or autoimmune CNS diseases in ~ 70% of patients. The CHANCE score could be of help to identify patients with high likelihood of BM and support clinical decision making.

## Introduction

Cerebrospinal fluid (CSF) analysis is an important tool to diagnose diseases of the central nervous system (CNS) [1]. Under normal conditions, the blood–brain barrier (BBB) limits immune cell trafficking into the CNS parenchyma to activated T cells, to ensure CNS immune surveillance. During neuroinflammation, immune cells can enter the CSF-filled subarachnoid space at the level of post-capillary venules and maybe via the choroid plexus and across the arachnoid barrier [2]. Elevated leukocyte count within the CSF is an indicator of neuroinflammation that can be caused by a variety of diseases [3], such as CNS infections, autoimmune diseases, trauma, degenerative processes and cancer. Yet, in up to 50% of patients, no etiology can be found [4]. In patients with CSF pleocytosis, the number of cells and the cell type determined by cytological examination already give an indication about the underlying cause. While a predominance of neutrophil granulocytes suggests bacterial meningitis, viral infections usually display a CSF enriched with lymphocytes and monocytes. In addition, the presence of erythrophages or siderophages indicates subarachnoid hemorrhage, whereas prevalence of tumor cells within the CSF is evidence of meningeal carcinomatosis [5].

Finding the correct diagnosis for a patient with elevated white cell count in the CSF can be a challenging task, but is vital for implementing the correct treatment. Acute bacterial meningitis is one of the most dangerous causes of acute neuroinflammation with a high mortality rate and high risk for long-term neurological sequelae [6]. Early diagnosis and prompt start of antibiotic treatment are indispensable for improving clinical outcomes [7].

Our study had two main objectives: (1) to characterize the various causes of CSF pleocytosis through an extensive analysis of a large patient population across all clinical departments, (2) to develop a scoring system capable of distinguishing bacterial meningitis from other diseases presenting with CSF pleocytosis.

## Patients and methods

A retrospective cohort study was performed including all in-patients with an elevated leukocyte count in their lumbar CSF—defined as > 5 cells/µl—from January 1st, 2015, to December 31st, 2017, in a large university hospital in Germany.

### Selection criteria

In total, 2387 CSF samples from 1289 adult (≥ 18 years) patients were identified to have increased CSF cell counts—out of 15,435 CSF samples totally tested in the same period. If multiple CSF analysis was performed for a single patient, only the initial CSF sample was used for analysis. CSF samples from external ventricular drains were excluded. Fifteen patients with incomplete medical records as well as 24 pediatric patients were excluded. To identify false positive cases because of a traumatic lumbar puncture, 1 leukocyte/µl was subtracted from the total CSF leukocyte count for every 1000 erythrocytes/µl. As a result, 1150 patients with elevated CSF cell count were included in this study.

### Data collection

The included patients were assessed for demographic data, clinical presentation, blood and CSF parameters, microbiological and pathological findings, diagnosis and clinical outcome. According to the diagnosis documented in the patients’ files, patients were allocated to disease categories: (1) CNS infections—with determined pathogen if a causative pathogen could be identified via microbiological testing vs. (2) CNS infections without determined pathogen if the treating physician diagnosed a CNS infection due to clinical and laboratory parameters, but no pathogen was identified microbiologically, (3) autoimmune CNS diseases, (4) neoplastic CNS diseases and (5) other diseases.

### Statistical analysis

Descriptive data are displayed as counts, percentage, median and range. Statistical analyses were performed using IBM SPSS Statistics version 26. Descriptive statistics for demographic, clinical and laboratory parameters were reported for each diagnostic category and subcategory. Univariate analyses were performed to detect a correlation between the presence of meningitis symptoms and the level of CSF pleocytosis. The ANOVA test with Bonferroni post hoc testing was used to determine significant differences in continuous parameters between diagnostic groups, while Chi-square tests were used for differences in binary variables. As CSF cell count data were not normally distributed according to Shapiro–Wilk test, Mann–Whitney tests and Kruskal–Wallis tests were used as nonparametric tests to confirm the results.

For the development of the CHANCE score, patients were separated into patients with acute bacterial meningitis (*n* = 38) and all other patients with elevated CSF cell count (*n* = 1112). Several binary criteria were tested for significant associations with acute bacterial meningitis using Chi-square tests. Afterward, significant criteria were used as independent variables in a binary logistic regression with bacterial meningitis as dependent variable. As a result, we created a score with the odds ratios of six identified criteria, calculated the score value for every patient and used them for receiver operating characteristic (ROC) analysis differentiating between bacterial meningitis and other causes of CSF pleocytosis. For validation, we used a cohort of 173 patients who underwent a lumbar puncture due to suspected CNS infection in our emergency room between January 2018 and November 2019 and showed an elevated CSF cell count > 5 cells/µl. The score was calculated for every patient of the validation cohort including 17 patients with acute bacterial meningitis and 156 other cases of CSF pleocytosis, resulting in another ROC analysis.

### Ethics and consent

This study was approved by the local ethical committee of LMU Munich (record no. 17-598).

## Results

### Etiologies of CSF pleocytosis

**Fig. 1:**
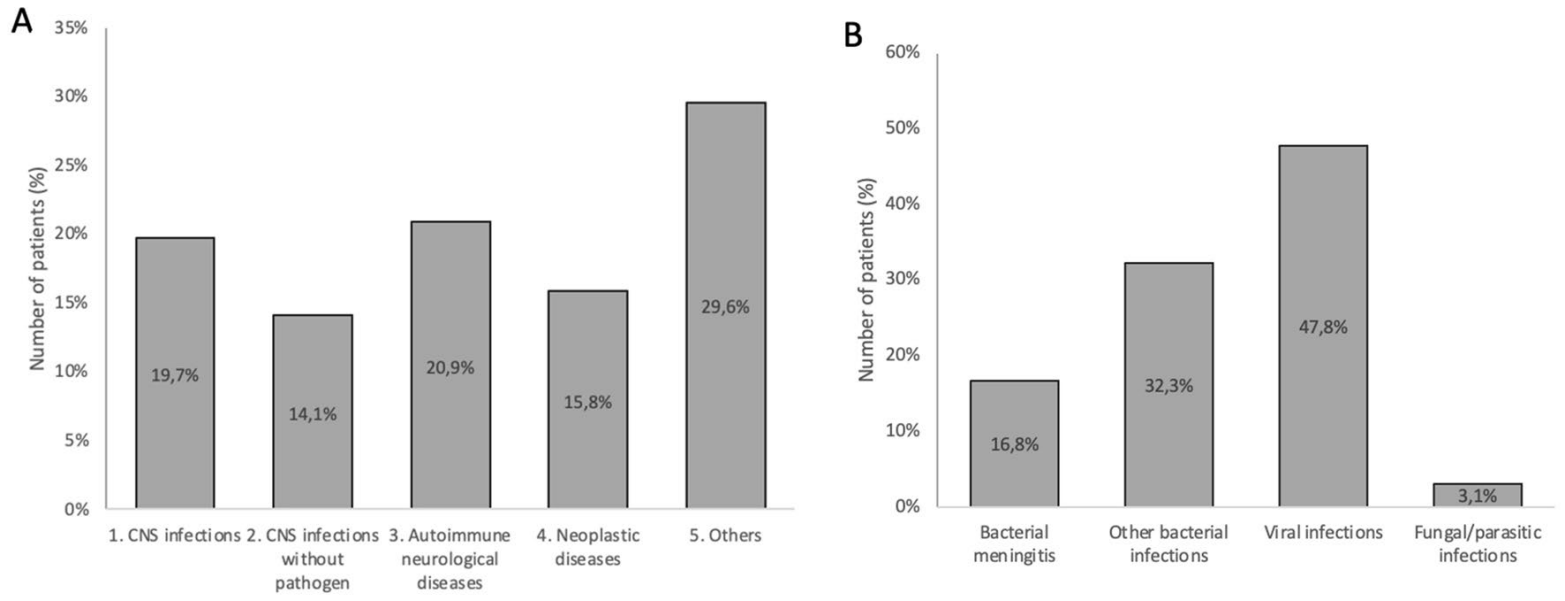
Etiologies of CSF pleocytosis. **A** Distribution of patients with CSF pleocytosis according to etiology. **B** Patients with CNS infections (1) were further divided according to causing pathogen

Among 1150 adult patients with CSF pleocytosis, 226 patients (19.7%) were diagnosed with CNS infections with a confirmed pathogen (Fig. 1). There were 38 cases of bacterial meningitis and 73 other acute or chronic bacterial infections which were defined as CNS infections caused by a bacterial pathogen except bacterial meningitis, such as Lyme neuroborreliosis, neurosyphilis, brain abscess, sepsis or ventriculitis. There were 108 viral CNS infections and 7 infections caused by other pathogens, namely *Candida albicans* (*n* = 1)*, Aspergillus fumigatus* (*n* = 3)*, Cryptococcus neoformans* (*n* = 2) and *Toxoplasma gondii* (*n* = 1). In another 162 patients (14.1%), CNS infections were diagnosed by the treating physician at that time based on clinical presentation and laboratory parameters, but no pathogen could be confirmed by microbiologic workup. Taken together, these two groups with a total of 388 patients—one-third of the whole collective—constituted the largest diagnostic group.

Another 240 patients (20.9%) with elevated CSF cell counts were grouped as autoimmune neurological diseases: the majority of these patients (*n* = 144) had multiple sclerosis, while the rest suffered from other inflammatory or autoimmune CNS diseases such as neurosarcoidosis, vasculitis, Guillain-Barré syndrome, autoimmune cerebellitis, myelitis or autoimmune encephalitis. In another 182 patients (15.8%) CNS pleocytosis was caused by neoplastic CNS diseases, which included patients with meningeal carcinomatosis (*n* = 104), cerebral lymphoma (*n* = 31) and other forms of cancer (*n* = 47), mostly brain metastases, meningioma, astrocytoma, glioma and other primary CNS tumors. The last category of 340 patients (29.6%) was labeled as “others,” with iatrogenic condition (*n* = 77), CNS hemorrhage (*n* = 33) and seizure (*n* = 33) being the most common etiologies (Table 1).

**Table 1:** Patient numbers and CSF cell count according to diagnostic groups

Diagnostic groups	Patient count	CSF cell count/µl: median (range)
1. CNS infections	226	82.5 (6–172,000)
(a) Bacterial meningitis^a^	38	2055 (25–172,000)
(b) Other bacterial infections	73	48 (6–20,490)
(c) Viral infections	108	63 (6–930)
(d) Fungal/parasitic infections	7	118 (6–2512)
2. CNS infections without pathogen	162	72 (6–11,823)
3. Autoimmune neurological diseases	240	11 (6–2144)
Multiple sclerosis	144	11 (6–286)
Other inflammatory diseases	96	13.5 (6–2144)
4. Neoplastic diseases	182	19 (6–3570)
Meningeal carcinomatosis	104	32 (6–3570)
Cerebral lymphoma	31	14 (6–425)
Other cancers	47	9 (6–214)
5. Others	340	11 (6–14,395)
Iatrogenic	77	28 (6–14,395)
Unknown etiology	49	10 (6–736)
Miscellaneous	46	8 (6–166)
Cerebral bleedings	33	30 (7–1192)
Seizures	33	10 (6–130)
Primary headaches/psychiatric disorders	26	6.5 (6–26)
Cranial nerve palsies	25	9 (6–60)
Ischemic stroke	24	8 (6–31)
CSF pressure disorders	16	7 (6–24)
Spinal canal stenosis/disc herniation	11	12 (8–29)
Total	1150	

Number of patients and CSF cell count (median, range) for each diagnostic group

^a^Pathogens detected in patients with bacterial meningitis: *S. pneumoniae* (15), *S. aureus* (5), *H. influenzae* (3), *N. meningitidis* (2), *L. monocytogenes* (2), *Staphylococcus haemolyticus* (2), *Enterococcus faecium* (2), *Pseudomonas aeruginosa* (2), *S. anginosus* (1), *Gram-positive cocci* (1), *E. coli* (1), *Staphylococcus epidermidis* (1), *S. salivarius* (1). Upon critical review of patient details, contamination due to the last two pathogens did not appear likely

### Clinical characterization

In terms of baseline patient characteristics, patients with autoimmune neurological diseases were significantly younger than those of all other groups. Additionally, there were more female patients in this group (61.7%), while both infectious groups contained more male patients (Table 2).

**Table 2:** Demographics by diagnostic category

Diagnostic group	Gender: male (%)	Age: median (range)
1. CNS infections	60.6	54.5 (18–88)
2. CNS infections without pathogen	59.3	50 (18–90)
3. Autoimmune neurological diseases	38.3	40.5 (18–86)*
4. Neoplastic diseases	47.8	59 (18–84)
5. Others	51.5	55 (18–90)
1. CNS infections		
(a) Bacterial meningitis	50.0	65.5 (21–85)
(b) Other bacterial infections	71.2	56 (19–88)
(c) Viral infections	54.6	48 (18–85)*
(d) Fungal/parasitic infections	100.0	51 (41–78)
		*vs. a/b

Statistic significances were calculated by one-way ANOVA with Bonferroni post hoc testing

*p < 0.05

Next, the patients were assessed for their clinical presentation. Fever and nuchal rigidity were significantly more often observed in CNS infections. On the other hand, patients with autoimmune diseases presented less frequently with altered mental status and headache, but more often with focal neurological deficits (Table 3). Altered mental status, fever and nuchal rigidity appeared significantly more often in patients with bacterial meningitis than in other subgroups of CNS infections with determined pathogen.

**Table 3:** Frequency of symptoms by diagnostic category

Diagnostic group	Altered mental status	Fever	Nuchal rigidity	Neurologic deficit	Headache
1. CNS infections	58/225 (25.8%)	70/222 (31.5%)*	44/221 (19.9%)*	117/214 (54.7%)	103/210 (49.0%)
2. CNS infections without pathogen	36/162 (22.2%)	66/158 (41.8%)*	32/160 (20.0%)*	46/160 (28.7%)	96/156 (61.5%)
3. Autoimmune neurological diseases	3/240 (1.3%)*	11/239 (4.6%)	4/239 (1.7%)	187/238 (78.6%)*	32/237 (13.5%)*
4. Neoplastic diseases	20/179 (11.2%)	12/179 (6.7%)	6/177 (3.4%)	94/177 (53.1%)	43/176 (24.4%)
5. Others	61/340 (17.9%)	15/334 (4.5%)	11/333 (3.3%)	171/323 (52.9%)	103/325 (31.7%)
	*vs. 1/2/4/5	*vs. 3/4/5	*vs. 3/4/5	*vs. 1/2/4/5	*vs. 1/2/5
1. CNS infections					
(a) Bacterial meningitis	24/38 (63.2%)*	23/36 (63.9%)*	21/36 (58.3%)*	20/34 (58.8%)	20/31 (64.5%)
(b) Other bacterial infections	16/73 (21.9%)	11/72 (15.3%)	6/72 (8.3%)	50/68 (73.5%)*	16/67 (23.9%)*
(c) Viral infections	17/107 (15.9%)	35/107 (32.7%)	17/106 (16.0%)	42/105 (40.0%)	63/105 (60.0%)
(d) Fungal/parasitic infections	1/7 (14.3%)	1/7 (14.3%)	0/7 (0.0%)	5/7 (71.4%)	4/7 (57.1%)
	*vs. b/c	*vs. b/c		*vs. a/c	*vs. a/c

A number of patients with indicated symptoms are displayed in comparison with total patient number of the diagnostic group as well as calculated percentage. Statistic significances were calculated by one-way ANOVA with Bonferroni post hoc testing

*p < 0.05

**Fig. 2:**
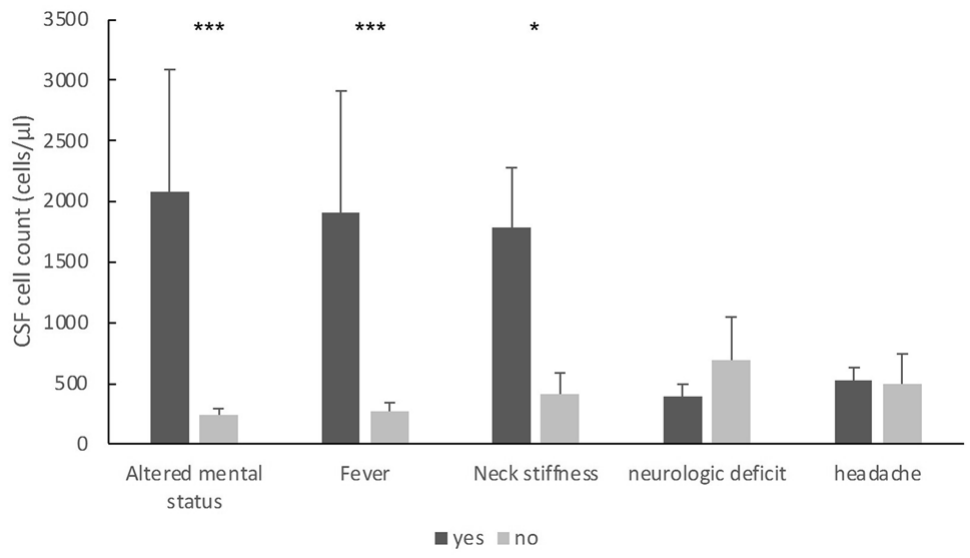
Level of CSF pleocytosis depending on the presence of symptoms. Values are given as mean + standard error of the mean. Significances were calculated by Student’s *t* tests. ***p < 0.001, **p < 0.01, *p < 0.05

CSF cell count was significantly higher in patients with altered mental status, fever or nuchal rigidity than in patients without these symptoms. Conversely, presence or absence of focal neurological deficits or headache did not correlate with greater CSF pleocytosis (Fig. 2).

A common constellation observed in the emergency room is a patient presenting with only headache and CSF pleocytosis, but without altered level of consciousness, fever, nuchal rigidity or focal neurological deficits. In this clinical situation, it is important to differentiate bacterial from viral CNS infection and rule out other severe differential diagnoses. We therefore analyzed patients with this specific constellation from our patient data and identified 125 such patients. Median follow-up was 7 days. Interestingly, not a single of these patients was diagnosed with acute bacterial meningitis. The most common diagnoses were viral meningitis, suspected viral meningitis without detected pathogen and primary headaches.

### Laboratory blood and CSF parameters

The patients’ blood was analyzed for their white blood cell (WBC) count and C-reactive protein (CRP), while their CSF was examined for their white cell count, protein level and glucose level. Glucose index was calculated from CSF glucose/blood glucose level.

Systemic inflammation, indicated by elevated white blood cell count and CRP level in the patients’ blood, was highest in patients of both infectious groups and lowest in patients with autoimmune neurological diseases (Table 4).

**Table 4:** Laboratory blood and CSF parameters depending on diagnostic category

(A) Systemic parameters				
Diagnostic group	WBC count (G/l)		CRP (mg/dl)	
1. CNS infections	8.3 (0.0–35.0)	214/226	1.2 (0.1–39.6)	214/226
2. CNS infections without pathogen	8.9 (1.2–33.5)	158/162	1.6 (0.1–36.6)	158/162
3. Autoimmune neurological diseases	7.2 (3.2–22.0)*	215/240	0.2 (0.1–25.0)*	205/240
4. Neoplastic diseases	7.3 (0.1–127.0)	156/182	0.6 (0.1–34.8)	154/182
5. Others	8.1 (1.6–41.6)	292/340	0.5 (0.1–31.0)	275/340
	*vs. 1./2.	**1035/1150**	*vs. 1./2./4./5.	**1006/1150**
1. CNS infections				
(a) Bacterial meningitis	14.3 (0.45–33.5)***		12.9 (0.1–39.6)***	
(b) Other bacterial infections	8.8 (2.7–35.0)		1.3 (0.1–37.1)	
(c) Viral infections	7.5 (0.9–15.8)		0.6 (0.1–13.7)	
(d) Fungal/parasitic infections	7.6 (0.0–10.4)		3.3 (0.2–7.4)	

(B) CSF parameters									
Diagnostic group	CSF cell count (/µl)	Neutrophilic^b^	Lymphocytic^b^	CSF protein (mg/dl)		CSF glucose (mg/dl)		Glucose index (CSF/blood)	
1. CNS infections	82.5 (6–172,000)*	56/226 (24.8%)	165/226 (73.0%)	85 (23–1513)*	223/226	58 (10–138)	223/226	0.54 (0.04–1.25)*	197/226
2. CNS infections without pathogen	72 (6–11,823)	42/162 (25.9%)	113/162 (69.8%)	75 (25–1939)*	161/162	58 (11–134)	161/162	0.54 (0.09–1.19)*	140/162
3. Autoimmune neurological diseases	11 (6–2144)^a^	9/240 (3.8%)	223/240 (92.9%)	48 (16–569)	240/240	61 (24–111)	238/240	0.62 (0.24–1.38)	224/240
4. Neoplastic diseases	19 (6–3570)	10/182 (5.5%)	134/182 (73.6%)	97 (25–1980)*	177/182	54 (10–286)	178/182	0.47 (0.08–0.92)*	101/182
5. Others	11 (6–14395)	63/340 (18.5%)	242/340 (71.2%)	58 (22–5195)	335/340	63 (10–170)*	336/340	0.60 (0.10–1.08)	251/340
	*vs. 3./4./5.			*vs. 3./5.	**1136/1150**	*vs. 1./2./4.	**1136/1150**	vs. 3./5.	**913/1150**
1. CNS infections									
(a) Bacterial meningitis	2055.5 (25–172000)***	29/38 (76.3%)	7/38 (18.4%)	407 (61–1513)***		26 (10–109)***		0.19 (0.04–0.59)***	34/38
(b) Other bacterial infections	48 (6–20490)	15/73 (20.5%)	57/73 (78.1%)	91 (23–424)		61 (10–138)		0.59 (0.26–1.25)	62/73
(c) Viral infections	63 (6–930)	11/108 (10.2%)	95/108 (88.0%)	71 (26–588)		60 (39–110)		0.56 (0.36–0.84)	96/108
(d) Fungal/parasitic infections	118 (6–2512)	1/7 (14.3%)	6/7 (85.7%)	98 (41–249)		46 (10–65)		0.28 (0.12–0.53)	5/7
	***vs. b/c					***vs. b/c		***vs. b/c	**197/226**

Total patient counts were marked in bold

(A) Systemic parameters: white blood cell (WBC) count, CRP. (B) CSF parameters: CSF cell count, cell cytology, CSF protein, CSF glucose and glucose index. Values are given as median (range). The second column shows the number of patients in which the parameters were available vs. total patient number of the diagnostic group. Statistic significances were calculated by one-way ANOVA with Bonferroni post hoc testing

*p < 0.05, ***p < 0.001

^a^One outlier with CSF cell count 2144/µl: patient with history of multiple ischemic events, no evident systemic signs of infection. Despite atypical CSF findings, an autoimmune etiology associated with small vessel vasculitis was postulated

^b^Neutrophilic pleocytosis was defined as > 50% neutrophils in cell cytology differentiation, lymphocytic pleocytosis as > 50% lymphocytes/monocytes

Regarding CNS inflammation, CSF cell count was higher in the group of CNS infections with detected pathogen (median 82.5 cells/µl) compared to autoimmune (11 cells/µl, *p* = 0.001), neoplastic (19 cells/µl, *p* = 0.01) and other causes of elevated CSF cell count (11 cells/µl, *p* < 0.001), but similar to patients with suspected CNS infections without detected pathogen (72 cells/µl, *p* = 0.057). Within the category of “other diseases,” patients with cerebral bleedings and iatrogenic conditions showed the highest median CSF cell counts (Table 1).

CSF protein levels were highly elevated in patients with neoplastic diseases (median 97 mg/dl) and both infectious groups (85 mg/dl [with pathogen] and 75 mg/dl [without pathogen]), while CSF glucose levels were lower in those patient groups. Of note, simultaneous serum glucose measurement (within 3 h) to calculate CSF/serum glucose index was determined in only 79% of all patients, while CSF protein and CSF glucose were measured in 99% of the cases.

Within the group of CNS infections with determined pathogen, the subgroup of patients with acute bacterial meningitis revealed the highest rates of CSF cell count, WBC count, CRP and CSF protein as well as predominantly neutrophilic cytology (Table 4). Patients with bacterial meningitis also showed significantly lower CSF glucose and CSF/blood glucose index as other bacterial or viral infections.

### Clinical outcome

Patients with neoplastic CNS diseases suffered from the highest mortality rate—16.6% compared to 0–5.8% in the other patient groups and had longer duration of hospital stay compared with patients of all other diagnostic categories (Table 5).

**Table 5:** Clinical outcome according to diagnostic category

Diagnostic group	Stay in hospital (days)	Stay in ICU (%)	Mortality rate (%)
1. CNS infections	10 (0–180)	60/226 (26.5%)*	13/225 (5.8%)
2. CNS infections without pathogen	10 (0–116)	47/162 (29.0%)*	7/161 (4.3%)
3. Autoimmune neurological diseases	5 (0–115)	8/240 (3.3%)	0/240 (0%)
4. Neoplastic diseases	14 (0–197)	11/182 (6.0%)	30/181 (16.6%)*
5. Others	7 (0–133)	62/340 (18.2%)*	9/340 (2.6%)
1. CNS infections			
(a) Bacterial meningitis	18.5 (1–92)	28/38 (73.7%)*	7/38 (18.4%)*
(b) Other bacterial infections	12 (0–111)	19/73 (26.0%)	5/73 (6.8%)
(c) Viral infections	7 (0–180)*	12/108 (11.1%)	1/107 (0.9%)
(d) Fungal/parasitic infections	36 (11–50)	1/7 (14.3%)	0/7 (0%)
			*vs. c

Stay in hospital is given in median days and range. Stay in ICU and mortality rate is calculated in percent of all patients within each group. Statistical analysis was calculated by one way-ANOVA with Bonferroni post hoc testing

*p < 0.05

Patients with CNS infections with or without determined pathogen as well as patients with other causes for CSF pleocytosis were significantly more frequently treated on ICU than patients with autoimmune or neoplastic diseases: 26.5%, 29.0% and 18.2%, respectively, compared to 3.3–6.0% in the other groups.

Within the group of patients with infectious diseases with determined pathogen, mortality rate (18.4%) was the significantly highest in patients with bacterial meningitis compared to other CNS infections. The duration of hospital stay was significantly lowest in patients with viral CNS infections, while patients with fungal or parasitic CNS infections stayed for a median of 36 days in hospital.

### CHANCE score for detecting bacterial meningitis

Lastly, we developed a diagnostic tool from the gathered data to differentiate between patients with and without bacterial meningitis.

Binary logistic regression on our patient collective identified six criteria to significantly differentiate between bacterial meningitis and other causes of CSF pleocytosis: CSF cell count > 100/µl (C), high CRP > 5 mg/dl (H), altered mental status (A), nuchal rigidity (N), CSF protein > 100 mg/dl (C) and elevated WBC count (E). CSF glucose and CSF/blood glucose index were not identified as relevant parameters for discrimination. Using the calculated odds ratio for each criterion, the following formula was created: BM = 7.8xC + 7.9xH + 3.5xA + 9.9xN + 11.4xC + 5.8xE. Using the scores of all patients, a ROC analysis differentiating between bacterial meningitis and all other patients revealed an AUC of 0.955, sensitivity of 92.1% and specificity of 90.9% at a cutoff value of 23.5. When comparing patients with bacterial meningitis and patients with viral CNS infections, AUC was 0.939.

We further validated the results of the score in a separate cohort of 173 patients with CSF pleocytosis who were admitted to the hospital via the emergency department from January 2018 to November 2019. In consequence to the circumstance that all patients in this validation cohort were admitted to the hospital as emergencies, the characteristics of the patients differed from the original derivation cohort in several aspects such as age and frequency of fever, nuchal rigidity, neurological deficits and headache (Table 6). The calculated CHANCE scores of these patients resulted in another ROC analysis with AUC of 0.956 (sensitivity 88.2%, specificity 87.2%).

**Table 6:** Patient characteristics in derivation and validation cohort

	Derivation (*n* = 1150)	Validation (*n* = 173)	*p* value
Age: median (range)	52 (18–90)	41 (18–93)	0.001
Gender: male	51.0%	50.3%	0.853
Altered mental status	15.5%	12.1%	0.245
Fever	15.4%	29.7%	0.001
Nuchal rigidity	8.6%	24.1%	0.001
Focal neurologic deficit	55.3%	24.5%	0.001
Headache	34.1%	67.9%	0.001
CSF cell count/µl	18 (6–172,000)	74 (6–14,839)	0.841
WBC count (G/l)	7.9 (0.0–127.0)	8.8 (2.3–38.4)	0.274
CRP (mg/dl)	0.6 (0.1–39.6)	0.6 (0.1–40.7)	0.241
CSF protein (mg/dl)	65 (16–5195)	64 (23–1000)	0.920
CSF glucose (mg/dl)	60 (10–286)	60 (10–158)	0.271
Glucose index	0.57 (0.04–1.38)	0.57 (0.05–1.02)	0.653

*p* value was calculated via unpaired *t* test or Chi-square test

## Discussion

CNS infections accounted for one-third of all cases with CSF pleocytosis, while autoimmune and neoplastic diseases were found in 21% and 16% of patients, respectively. Thirty percentages were due to “other diseases.” This underscores the importance of CNS infections as the cause of CSF pleocytosis on the one hand; on the other hand, it also demonstrates the necessity to think of alternative etiologies in the majority of patients who are found to have CSF pleocytosis.

A smaller study which examined 244 patients with elevated CSF cell count > 5 cells/µl found infectious causes in 18%—comparably to the percentage of patient with CNS infections with determined pathogen of 19.7% in our study. The number of patients with neoplastic (11%) as well as inflammatory (5%) and autoimmune diseases (4%) was lower than in our study [4]. In contrast, 53% of the cases were initially reported as unknown. Of those, 79 patients were treated for a presumptive diagnosis with the majority being suspected CNS infections (51/244, 20.9%)—correlating with our 14.1% of patients with CNS infections without determined pathogen. Another 51 patients (20.9%) of the category “unknown” remained undiagnosed. In our study, only 49 cases (4.3%) were reported as unknown reflecting the differences in diagnostic rigor of CSF pleocytosis. Interestingly, in another Danish study on the etiologies of patients with CSF pleocytosis, a much higher percentage of infections (61.4%) was reported followed by miscellaneous causes (12.7%), vascular (9.7), neurodegenerative (7%), neoplastic (5%) and inflammatory causes (4.2%) [8]. This discrepancy is mostly due to a different cell count cutoff: The Danish study included patients with > 10 cells/µl CSF, while this study included those with > 5 cells/µl, resulting in a higher percentage of patients with CNS infections in the Danish cohort. When using the same cutoff of 10 cells/µl CSF in our patient collective, CNS infections (*n* = 199, 24.4% [with detected pathogen] and *n* = 136, 16.7% [without pathogen]) comprised 41.1% of all cases, while autoimmune (*n* = 153), neoplastic (*n* = 135) and other CNS diseases (*n* = 192) contributed 17% and 24%. The Danish study analyzed patient data through the biochemical database from 5 hospitals in the capital region of Denmark from 2003 to 2010. Further differences in frequency of the various CNS diseases might be due to differences in patient cohorts or hospital care levels.

Regarding meningitis, CSF white cell count is a well-known marker that can help differentiate between bacterial and viral origin: One study reported that 99% of patients with bacterial meningitis show CSF cell count over 100 cells/µl, while 87% exceed 1000 cells/µl [9]. Yet, in another cohort study, only 66% of patients with bacterial meningitis revealed a white cell count over 1000/µl [10], thus rendering CSF cell count solely not a reliable predictive factor. A typical constellation with granulocytic pleocytosis > 1000 cells/µl, CSF protein > 1000 mg/l and lactate > 3.5 mmol/l is present in approximately 80% of cases [1]. Other parameters such as CSF lactate, serum procalcitonin and CSF glucose [11] have been suggested previously as predictive of bacterial meningitis with mixed results of sensitivity and specificity [12–15]. Even though predictive factors indicating bacterial meningitis have been identified, highly reliable markers are still lacking.

The values of blood and CSF glucose to calculate glucose index were determined simultaneously (within 3 h) in close to 80% of our patient cohort. Interestingly, in patients that were treated primarily in the department of neurology, the CSF/serum glucose index was calculated in over 90% of their patients—as it is standard there—while in patients treated in non-neurological departments, a CSF/serum glucose index was available in only ~ 50% of cases. These real-world data represent an important finding showing there is still room for improvement in CSF examination, especially in non-neurological departments. A replacement of the standard glucose index by the determination of CSF lactate could be a solution. The incomplete determination of glucose index in patients with CSF pleocytosis might have contributed to the absence of significance for differentiating between bacterial meningitis and other causes of elevated CSF count. Another reason might be the presence of e.g., patients with meningeal carcinomatosis and thus reduced glucose index within the control group. In line, when we tried to differentiate bacterial meningitis from viral infections, the glucose index was highly significant (0.19 vs. 0.56, *p* < 0.001), correlating with previous studies that reported significantly lower glucose index in patients with bacterial meningitis than in those with viral meningitis [14, 15]. Several recent studies proposed CSF lactate as most accurate parameter to discriminate bacterial from viral meningitis [12, 16, 17], attributing it higher diagnostic accuracy than CSF glucose, glucose quotient, CSF protein and CSF leukocyte count [18]. As of late, CSF lactate is increasingly used to discriminate CSF infections from non-infectious causes of CSF abnormalities in post-neurosurgical patients [19, 20] although it is not sufficient as a single marker [21–23]. Yet, as CSF lactate parameters were not available for all patients in our study due to the need of an extra sample for its determination, it was not included in the CHANCE score. Moreover, as elevated CSF lactate occurs in various neurological diseases including stroke, seizures and mitochondrial pathologies [7, 24, 25], differentiating bacterial meningitis from other causes of CSF pleocytosis might be less accurate than in comparison with viral meningitis.

Regarding the etiology of CSF pleocytosis, the last group of CNS diseases “other” than infectious, autoimmune or neoplastic causes, represents a group of great clinical interest. There were 30% miscellaneous CNS diseases such as cerebral hemorrhage, ischemic stroke or epilepsy in our study. In some of these cases, for example patients with primary headaches, psychiatric disorders, ischemic strokes or seizures, the documented diagnoses do not explain the CSF pleocytosis. This discrepancy might be due to the retrospective design of this study where re-testing was not possible. In some cases, there could have been a coincidence of the documented patient’s diagnosis and a neuroinflammatory reaction with undetected etiology. Initially, there were 30 patients with neurodegenerative disorders and CSF pleocytosis. When analyzed in detail, we found the following main explanations for CSF pleocytosis: (1) contamination of CSF with peripheral blood or bone marrow and placement of a Tuohy drain before CSF analysis in cases of normal pressure hydrocephalus. Also, there are several documented cases in the literature of patients with seizures or ischemic strokes who concomitantly show elevated CSF cell count [26–29]. Other studies have reported patients with CSF pressure disorders, post-intracranial surgery or even depression to display CSF pleocytosis [30–34]. Therefore, in most cases of our miscellaneous group, elevated CSF cell count was a secondary finding without relevance for the patients’ primary disease. The group of 47 patients with “other neoplastic diseases” and CSF pleocytosis are not unexpected as our clinic is a large center for hematooncology and neurosurgery where many patients with brain tumors are treated who can present with elevated cell count in up to 30% of cases [35].

Assessing a combination of clinical features and laboratory parameters, we tried to develop a score to differentiate bacterial meningitis from other disease entities with CSF pleocytosis. The score was developed from the clinical data of our cohort and revealed that the presence of altered mental status, nuchal rigidity, CSF cell count > 100 cells/µl, CSF protein > 100 mg/dl, elevated white blood cell count and CRP > 5 mg/dl indicate bacterial meningitis in patients with CSF pleocytosis. For the score to reach the cutoff, 4 or more of those criteria must be fulfilled. Therefore, when a patient with elevated CSF cell count shows 4 or more of those criteria, bacterial meningitis is very likely. Still, sensitivity of 92% and specificity of 91% are not sufficient alone for clinical decision making and to rule out bacterial meningitis based on the score alone. As such, it is still important to start empiric antibiotic therapy as early as possible in cases with suspected bacterial meningitis, even if single characteristic symptoms are missing. This can especially be the case in immunosuppressed patients who are less likely to present with characteristic meningitis symptoms [36]. However, the CHANCE score can, among others, still be a helpful additional tool in clinical decision making,

Other scores for adults included CSF pleocytosis, CSF gram stain and abnormal immune status, examination or laboratory findings [37], CSF cell count, CSF protein, CSF lactate, CSF/serum glucose index and peripheral neutrophil count [38, 39]. Overall, several factors of other scores differentiating bacterial meningitis are also included in our CHANCE score.

In contrast to existing scores, the CHANCE score was developed from a large patient cohort for adults to discriminate bacterial meningitis not only against viral meningitis, but also against all other causes of CSF pleocytosis. The containing clinical and laboratory criteria can be gained very quickly, even at night, and give an indication about probability of bacterial meningitis without further microbiological testing.

A limitation of this study lies in its retrospective character which did not allow for retesting of the documented diagnoses. In several miscellaneous cases, CSF pleocytosis might be a secondary diagnostic finding not connected with the patients’ primary disease. Moreover, for the development of the CHANCE score, only factors that were examined in most patients could be evaluated, leading to exclusion of CSF lactate, for example.

In conclusion, this study illustrates the most common causes for CSF pleocytosis including CNS infections, neoplastic or autoimmune CNS diseases. We identified the following criteria as relevant for differentiating cases of bacterial meningitis from other causes of CSF pleocytosis: CSF cell count > 100 cells/µl, CRP > 5 mg/dl, altered mental status, nuchal rigidity, CSF protein > 100 mg/dl and elevated WBC count.

## Data Availability

The data that support the findings of this study are available from the corresponding author upon reasonable request.
